# Use of Genome-Wide Association Studies for Cancer Research and Drug Repositioning

**DOI:** 10.1371/journal.pone.0116477

**Published:** 2015-03-24

**Authors:** Jizhun Zhang, Kewei Jiang, Liang Lv, Hui Wang, Zhanlong Shen, Zhidong Gao, Bo Wang, Yang Yang, Yingjiang Ye, Shan Wang

**Affiliations:** 1 Department of Gastroenterological Surgery, Peking University People’s Hospital, Beijing, 100044, PR China; 2 Department of Clinical Laboratory, Peking University People’s Hospital, Beijing, 100044, PR China; Tor Vergata University of Rome, ITALY

## Abstract

Although genome-wide association studies have identified many risk loci associated with colorectal cancer, the molecular basis of these associations are still unclear. We aimed to infer biological insights and highlight candidate genes of interest within GWAS risk loci. We used an *in silico* pipeline based on functional annotation, quantitative trait loci mapping of cis-acting gene, PubMed text-mining, protein-protein interaction studies, genetic overlaps with cancer somatic mutations and knockout mouse phenotypes, and functional enrichment analysis to prioritize the candidate genes at the colorectal cancer risk loci. Based on these analyses, we observed that these genes were the targets of approved therapies for colorectal cancer, and suggested that drugs approved for other indications may be repurposed for the treatment of colorectal cancer. This study highlights the use of publicly available data as a cost effective solution to derive biological insights, and provides an empirical evidence that the molecular basis of colorectal cancer can provide important leads for the discovery of new drugs.

## Introduction

Since the advent of high-density single nucleotide polymorphism (SNP) genotyping arrays, researchers have used genome-wide association studies (GWAS) to identify innumerable loci associated with a multitude of diseases. The vast majority of SNPs identified by GWAS are within the intergenic or intronic regions (approximately 88%)[1,2]. GWAS has also enabled the discovery of many genetic variations of colorectal cancers (CRC). The next step was to identify the genes that were affected by causal variants, which would enable us to translate the risk SNPs to meaningful insights on pathogenesis.

Most reports have simply implicated the nearest gene to a GWAS hit as a target of the functional variant without any evidence[1]. The identification of expression quantitative trait loci (eQTL) has been proposed as a promising method to find the candidate genes associated with a disease risk[3][4]. It should be noted that identifying an eQTL provides only an indirect evidence of a link between genotype and gene transcription [1].

As far as we know, there’s simply no good way to identify these target genes, which is key to understanding the mechanism by which GWAS variants act. So we proposed a bioinformatics pipeline to prioritize the most likely candidate genes by using several biological data sets. Seven criteria were adopted to prioritize candidate genes. The widely used eQTL criterion mentioned above is only one of seven criteria in the pipeline.

One way of accelerating the translation of data from GWAS into clinical benefits, is to use the results to identify new indications for treatment with existing molecules. GWAS may be used to construct drug-related networks, aiding drug repositioning. Although GWAS do not directly identify most of the existing drug targets, there are several reasons to expect that new targets will nevertheless be discovered using these data[5,6]. Initial results on drug repurposing studies using network analysis are encouraging and suggest directions for future development[7]. By integrating rheumatoid arthritis genetic findings with the catalog of approved drugs for rheumatoid arthritis and other diseases, Okada Y et al provided an empirical data to indicating that genetic approaches may be useful for supporting genetics-driven genomic drug discovery efforts in complex human traits[8].

In the present study, we used the *in silico* pipeline to systematically integrate data on risk loci for CRC biology and drug discovery from a variety of databases.

## Materials and Methods

**Fig 1:**
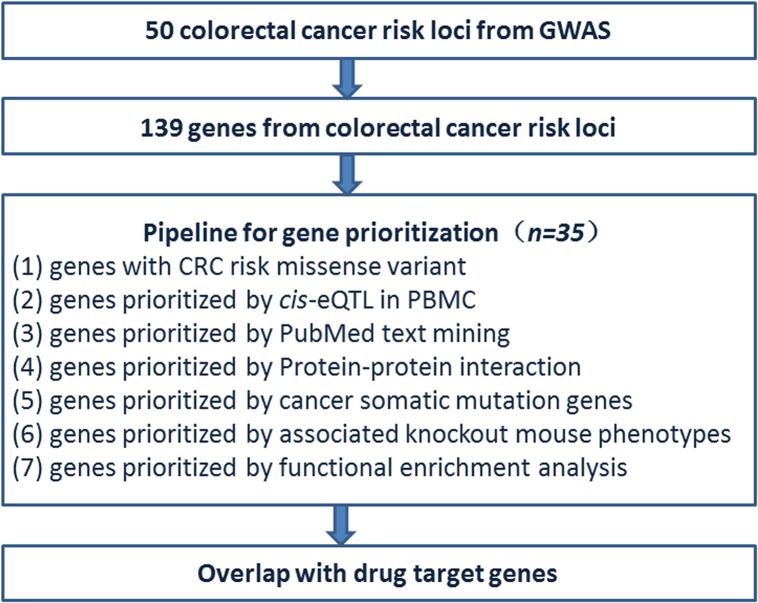
An overview of the study design. One hundred and forty-seven candidate genes were obtained from 50 CRC risk loci. A bioinformatics pipeline was developed for the prioritization of these candidate genes. Seven criteria were used to score the genes: (1) CRC risk missense variant; (2) *cis*-eQTL; (3) PubMed text mining; (4) PPI; (5) cancer somatic mutation; (6) knockout mouse phenotype; and (7) functional enrichment. Extent of overlap with target genes for approved CRC drugs was also assessed.

An overview of the study design is illustrated in Fig. 1. Biological candidate genes were obtained from GWAS-identified CRC risk loci. Next, the genetic data were integrated with the results of statistical analyses, computational approaches, and publicly available large data sets to prioritize the obtained genes, and propose new targets for drug treatments.

### CRC risk loci from GWAS

We downloaded CRC risk SNPs from the National Human Genome Research Institute (NHGRI) GWAS catalogue database on January 31, 2014 [2].

### Biological candidate genes from CRC risk loci

It is a well-known fact that the risk SNPs indicates haplotypes on which the functional variants reside; therefore, the next step was to identify their target genes. By adopting multi-annotations between risk SNPs and their surrounding genes, the *snp2gene* allowed conventional annotation due to their proximity, as well as linkage disequilibrium[9].

For each of the GWAS SNPs involved, we used the *snp2gene* to identify the candidate genes. For each gene in the risk loci, we evaluated if the gene was the nearest gene to the CRC risk SNP within the risk locus.

### Prioritization of candidate genes

By using several biological data sets, we devised a bioinformatics pipeline to prioritize the most likely candidate genes.

Firstly, functional annotations for CRC risk SNPs were identified by *ANNOVAR* [10]. Trait-associated variants were enriched within chromatin marks, particularly in H3K4me3[11]. H3K4me3 data of 34 cell-types could provide the fine mapping of associated SNPs to identify causal variation in the previous studies[12]. So we evaluated whether the CRC risk SNPs and SNPs in the linkage disequilibrium (r^2^ > 0.80) were overlapping with H3K4me3 peaks of 34 cell types. The H3K4me3 data were obtained from the National Institutes of Health Roadmap Epigenomics Mapping Consortium, by a permutation procedure with 10^5^ iterations [12]. We identified genes for CRC risk SNPs or SNPs from linkage disequilibrium (r^2^ > 0.80) that were annotated as missense variants.

Secondly, we assessed the *cis*-expression quantitative trait loci (*cis*-eQTL) effects using the data of 5,311 European subjects from the study on peripheral blood mononuclear cells (PBMCs) [13]. Westra HJ et al. had made a browser available for all significant *cis*-eQTLs, detected at a false-discovery rate of 0.50. (http://genenetwork.nl/bloodeqtlbrowser/) In their study, eQTLs were deemed *cis*-eQTLs when the distance between the SNP chromosomal position and the probe midpoint was less than 250 kb. The eQTLs were mapped using Spearman’s rank correlation on imputed genotype dosages. Resultant correlations were then converted to P values, and their respective z scores were weighted by the square root of sample size.

To evaluate *cis*-eQTL genes of risk SNPs, it was only needed to provide all risk SNP. When the CRC risk SNP was not available in eQTL data sets, we alternatively used the results of best proxy SNPs in linkage disequilibrium with the highest r^2^ value (r^2^ > 0.80).

Thirdly, by using the Gene Relationships among Implicated Loci (*GRAIL*), we evaluated the degree of relatedness among the genes within disease regions. *GRAIL* is a tool to examine the relationships between genes in different disease associated loci. Given many genomic regions or SNPs associated with CRC, *GRAIL* searches for similarities in the published scientific text among the associated genes[14]. A p value of <0.05 was considered significant. To avoid publications that reported on or were influenced by the disease regions discovered in the recent scans, we use only those PubMed abstracts published prior to December 2006, before the recent onslaught of GWA papers identifying novel associations, avoid any strong bias towards the genes closest to the associated SNPs. This approach effectively avoids this problem.[14].

Next, we used the Disease Association Protein-Protein Link Evaluator *(DAPPLE)* for assessing the presence of significant physical connectivity among proteins encoded by candidate genes by protein-protein interaction (PPI), reported in the literature. The *DAPPLE* takes a list of seed SNPs that converts them into genes based on the overlap. The hypothesis behind DAPPLE is that a genetic variation affects a limited set of underlying mechanisms that are detectable by PPI [15]. A p value of <0.05 was considered significant.

Nest, we obtained cancer somatic mutation genes from the Catalogue of Somatic Mutations in Cancer (*COSMIC*) database[16], and downloaded knockout mouse phenotype labels and gene information from the Mouse Genome Informatics (MGI) database[17] on April 8, 2014. We defined all CRC risk genes included in the CRC risk loci, and evaluated the overlap with cancer phenotypes with registered somatic mutations, and phenotype labels of knockout mouse genes with human orthologous. Hypergeometric distribution test was used for overlap statistical analyses with significance at a p value of <0.05.

Finally, we performed function enrichment analysis to investigate if genes affected by SNPs were enriched for specific functional categories or pathways. The DAVID Bioinformatics Resources that included Gene Ontology (GO), Kyoto Encyclopedia of Genes and Genomes (KEGG) and Online Mendelian Inheritance in Man (OMIM) were used for analyses.[18] The obtained results were considered significant at a *p* value of <0.05.

We scored each of the genes by using the following selection criteria, and calculated the number of the satisfied criteria: (1) genes with missense variants; (2) *cis*-eQTL genes of risk SNPs; (3) genes prioritized by PubMed text mining; (4) genes prioritized by PPI network; (5) cancer somatic mutation genes; (6) genes prioritized by associated knockout mouse phenotypes; and (7) genes prioritized by functional enrichment analysis.

Correlations of candidate gene prioritization criteria were evaluated by the Pearson correlation analysis. Each gene was scored based on the number of criteria that were met (scores ranged from 0–7 for each gene) in case of weak correlations. Genes with a score of ⩾2 were defined as ‘biological risk genes’.

### Drug validation and discovery

If human genetics can validate drug targets, then it can be used to identify whether the approved drugs currently used for treating other indications can be used for the treatment of CRC. We present here an analysis of the potential application of GWAS data, drug repositioning.

We obtained drug target genes and corresponding drug information from Drug Bank [19] and Therapeutic Targets Database (TTD)[20] on Oct 18, 2013. We selected drug target genes that had pharmacological activities and were effective in human orthologous models, and those which annotated with any of the approved, clinical trial or experimental drugs.

The drug target genes annotated to CRC drugs were manually extracted by professional oncologists. To decrease the rates of the false positive, only the drugs under current clinical use were involved in the study, which could be found in the NCCN clinical practice guidelines in colorectal cancers.[21]

We extracted genes from direct PPI with biological CRC risk genes by using protein interaction network analysis-2 (*PINA2)*, which integrates six well-known manually curated PPI databases[22].

We evaluated the possibility of exploring protein products from the identified biological risk genes, or any genes from a direct PPI network as targets of approved CRC drugs or drugs for other indications. Let x be the set of the biological CRC risk genes and genes in direct PPI with them (n_x_ genes), y be the set of genes with protein products that are the direct target of approved CRC drugs (n_y_ genes), and z be the set of genes with protein products that are the direct target of all approved drugs (n_z_ genes). We defined n_x∩y_ and n_x∩z_ as the numbers of genes overlapping between x and y and between x and z, respectively. Hypergeometric distribution tests were used for overlap statistical analyses, and a p value of <0.05 was considered statistically significant.

## Results

In the present study, 50 CRC-associated SNPs were obtained from NHGRI (Table 1), and 140 genes were obtained based on proximity and linkage disequilibrium using the *snp2gene* (S1 Table).

**Table 1:** Summary of 50 colorectal cancer GWAS risk alleles obtained from National Human Genome Research Institute.

**Risk SNPs**	**Region**	**p-Value**	**OR**	**95% CI**
rs10505477	8q24.2	9E-7	1.13	[1.08-1.19]
rs6983267	8q24.2	8E-28	1.2	[1.16-1.24]
rs4939827	18q21.1	1E-9	1.06	[1.03-1.09]
rs10795668	10p14	4E-6	1.27	[1.14-1.40]
rs16892766	8q23	2E-7	1.14	[1.08-1.19]
rs3802842	11q23.1	8E-7	1.18	[1.11-1.27]
rs4779584	15q13	1E-7	1.14	[1.08-1.18]
rs4939827	18q21.1	8E-9	1.28	[1.18-1.39]
rs4939827	18q21.1	2E-8	1.18	[1.11-1.25]
rs6983267	8q24.2	6E-10	1.11	[1.08-1.15]
rs7014346	8q24.2	3E-13	1.12	[1.10-1.16]
rs10411210	19q13.1	2E-7	1.14	[1.06-1.19]
rs4444235	14q22	7E-10	1.07	[1.04-1.10]
rs961253	20p12	1E-14	1.27	[1.16-1.39]
rs9929218	16q22	3E-11	1.17	[1.12-1.23]
rs10936599	3q26.2	4E-8	1.35	[1.20-1.49]
rs11169552	12q13.1	3E-6	1.47	[1.25-1.72]
rs4925386	20q13.3	4E-7	1.24	[1.14-1.34]
rs6687758	1q41	2E-10	1.12	[1.08-1.16]
rs6691170	1q41	1E-8	1.1	[1.06-1.12]
rs6983267	8q24.2	9E-26	1.19	[1.15-1.23]
rs7758229	6q25.3	7E-11	1.24	[1.17-1.33]
rs11632715	15q13.3	3E-6	1.37	[1.20-1.56]
rs16969681	15q13.3	9E-7	1.13	[1.08-1.19]
rs1957636	14q22.2	2E-10	1.12	[1.09-1.16]
rs16892766	8q23	4E-10	1.17	[1.11-1.22]
rs3802842	11q23.1	3E-6	1.13	[1.08-1.20]
rs4779584	15q13	2E-10	1.12	[1.18-1.16]
rs4939827	18q21.1	6E-6	1.11	[1.06-1.15]
rs7315438	12q24.2	3E-18	1.27	[1.20-1.34]
rs1321311	6p21.2	1E-10	1.11	[1.08-1.15]
rs3824999	11q13.4	1E-10	1.1	[1.07-1.13]
rs5934683	Xp22.2	2E-10	1.09	[1.05-1.11]
rs7972465	12q13.13	8E-7	1.18	[1.11-1.27]
rs10911251	1q25	2E-6	1.28	[1.16-1.43]
rs11903757	2q32.3	3E-6	1.06	[0.88-1.29]
rs13130787	4q22	3E-8	1.09	[1.06-1.13]
rs17094983	14q23	1E-11	1.13	[1.09-1.18]
rs1912453	1q23	5E-7	1.12	[1.08-1.19]
rs2057314	6q22.1	7E-6	1.1	[1.05-1.14]
rs2128382	8q24.2	4E-8	1.16	[1.10-1.22]
rs3217810	12p13.3	9E-6	1.07	[1.04-1.11]
rs3217901	12p13.3	3E-7	1.09	[1.06-1.13]
rs3802842	11q23.1	8E-6	1.11	[1.06-1.16]
rs4779584	15q13	5E-8	1.18	[1.11-1.25]
rs4813802	20p12	2E-8	1.18	[1.11-1.24]
rs4939827	18q21.1	4E-7	1.14	[1.08-1.20]
rs59336	12q24.2	3E-8	1.04	[1.04-1.10]
rs6983267	8q24.2	8E-10	1.11	[1.08-1.15]
rs10774214	12p13.3	2E-6	1.28	[1.15-1.41]
rs10774214	12p13.3	3E-6	1.28	[1.12-1.42]
rs1665650	10q25.3	5E-10	1.17	[1.11-1.23]
rs2423279	20p12	6E-8	1.2	[1.12-1.28]
rs647161	5q31.1	2E-9	1.09	[1.06-1.12]
rs10114408	9q22.3	5E-10	1.17	[1.11-1.23]
rs10879357	12q21.1	4E-6	1.27	[1.15-1.41]
rs17730929	4q13.2	4E-7	1.11	[1.06-1.15]
rs367615	5q21	3E-6	1.08	[1.04-1.11]
rs39453	7p15.3	4E-10	1.08	[1.05-1.10]
rs4591517	3p24.3	1E-9	1.08	[1.06-1.11]
rs9365723	6q25.3	1E-12	1.16	[1.09-1.27]
rs12548021	8p12	3E-6	1.25	[1.14-1.39]
rs3104964	8q22.1	4E-7	1.09	[1.06-1.13]
rs8180040	3p21.3	5E-7	1.23	[1.14-1.34]

### Functional annotations of CRC risk SNPs

Most SNPs (62%) were located in the intergenic regions (S2 Table). Two SNPs were identified in linkage disequilibrium with missense SNPs (r^2^ > 0.80; S3 Table). Next, we assessed 50 CRC risk loci for enriched epigenetic chromatin marks [12]. Of the 34 cell types investigated, we observed a significant enrichment of CRC risk alleles with H3K4me3 peaks in rectal mucosa cells (*p* = 0.00014 and 0.00023, respectively) (S4 Table).

### Cis-expression quantitative trait loci (*cis*-eQTL)

Using the *cis*-eQTL data obtained from the PBMC study [13], we found that 13 risk SNPs showed *cis*-eQTL effects (p < 0.0016 and FDR < 0.5) (S5 Table).

### PubMed text-mining

Twenty-four genes were prioritized based on data obtained by PubMed text mining using *GRAIL* with gene-based p < 0.05 [14] (S6 Table).

### Protein-protein interaction (PPI)

Two genes were prioritized by PPI network using gene-based *DAPPLE* with p < 0.05 [15] (S7 Table).

### Cancer somatic mutation

Among the 522 genes with registered somatic mutations obtained from the COSMIC database[16], a significant overlap was observed in genes associated in non-hematological cancers (5/6, P = 2.41E -05) (S8 Table).

### Knockout mouse phenotype

We evaluated overlap with genes implicated in knockout mouse phenotypes[17]. Among the 30 categories of phenotypes, we observed nine categories significantly enriched with CRC risk genes (p < 0.05), led by craniofacial phenotype (S9 Table).

### Functional enrichment analysis

GO analysis indicated that a few genes were enriched in three categories (S10 Table); two with KEGG pathways observed in cancer (p = 0.002), and one with small cell lung cancer (p = 0.011) were functionally related (S11 Table). Functional analysis by OMIM demonstrated enriched gene sets in colorectal diseases (S12 Table).

Based on these new findings, we adopted the following seven criteria to prioritize each of the 140 genes from the 50 CRC risk loci: (1) genes with CRC risk missense variant (n = 2); (2) *cis*-eQTL genes (n = 13); (3) genes prioritized by PubMed text mining (n = 24); (4) genes prioritized by PPI (n = 2); (5) cancer somatic mutation genes (n = 6); (6) genes prioritized by associated knockout mouse phenotypes (n = 40); and (7) genes prioritized by functional enrichment analysis (n = 31).

Because these criteria showed weak correlations with each other (R^2^ < 0.48; S13 Table), each gene was scored based on the number of criteria that were met (scores ranged from 0–7 for each gene).

Thirty-five genes (25.2%) had a score>2, which were defined as ‘biological risk genes’ (S1 Fig.). Three loci included multiple biological CRC risk genes, (for example, ROS1 and GOPC by rs2057314) (Table 2).

**Table 2:** Biological genes in the CRC risk loci with a score≥2.

SNP	gene	Nearest gene from risk SNP	missense variant	cis-eQTL	pubmed text-mining	PPI	cancer somatic mutation genes	knockout mouse phenotype	function	score
**rs3217810**	**CCND2**	**1**	**-**	**1**	**1**	**-**	**1**	**1**	**1**	**5**
**rs10774214**	**CCND2**	**1**	**-**	**-**	**1**	**-**	**1**	**1**	**1**	**4**
**rs1321311**	**CDKN1A**	**1**	**-**	**1**	**1**	**-**	**-**	**1**	**1**	**4**
**rs4444235**	**BMP4**	**1**	**-**	**1**	**1**	**-**	**-**	**1**	**1**	**4**
**rs9929218**	**CDH1**	**1**	**-**	**-**	**1**	**-**	**1**	**1**	**1**	**4**
**rs10114408**	**BARX1**	**1**	**-**	**-**	**1**	**-**	**-**	**1**	**1**	**3**
**rs10411210**	**RHPN2**	**1**	**-**	**-**	**1**	**-**	**-**	**1**	**1**	**3**
**rs10911251**	**LAMC1**	**1**	**-**	**-**	**1**	**-**	**-**	**1**	**1**	**3**
**rs10911251**	**LAMC2**	**-**	**-**	**-**	**1**	**-**	**-**	**1**	**1**	**3**
**rs13130787**	**ATOH1**	**1**	**-**	**-**	**1**	**-**	**-**	**1**	**1**	**3**
**rs39453**	**CYCS**	**1**	**-**	**1**	**-**	**-**	**-**	**1**	**1**	**3**
**rs4813802**	**BMP2**	**1**	**-**	**-**	**1**	**-**	**-**	**1**	**1**	**3**
**rs4925386**	**LAMA5**	**1**	**-**	**1**	**1**	**-**	**-**	**-**	**1**	**3**
**rs4939827**	**SMAD7**	**1**	**-**	**-**	**1**	**-**	**-**	**1**	**1**	**3**
**rs59336**	**TBX3**	**1**	**-**	**-**	**1**	**-**	**-**	**1**	**1**	**3**
**rs647161**	**PITX1**	**-**	**-**	**-**	**1**	**-**	**-**	**1**	**1**	**3**
**rs7315438**	**TBX3**	**1**	**-**	**-**	**1**	**-**	**-**	**1**	**1**	**3**
**rs11169552**	**ATF1**	**-**	**-**	**-**	**-**	**-**	**1**	**1**	**-**	**2**
**rs11632715**	**GREM1**	**-**	**-**	**-**	**1**	**-**	**-**	**-**	**1**	**2**
**rs12548021**	**DUSP4**	**1**	**-**	**-**	**-**	**-**	**-**	**1**	**1**	**2**
**rs16969681**	**GREM1**	**-**	**-**	**-**	**1**	**-**	**-**	**-**	**1**	**2**
**rs17094983**	**DACT1**	**1**	**-**	**-**	**1**	**-**	**-**	**1**	**-**	**2**
**rs1912453**	**RGS4**	**-**	**-**	**-**	**-**	**-**	**-**	**1**	**1**	**2**
**rs1957636**	**CDKN3**	**1**	**-**	**-**	**1**	**-**	**-**	**-**	**1**	**2**
**rs2057314**	**ROS1**	**-**	**-**	**-**	**-**	**-**	**1**	**1**	**-**	**2**
**rs2057314**	**GOPC**	**1**	**-**	**-**	**-**	**-**	**1**	**-**	**1**	**2**
**rs367615**	**MAN2A1**	**1**	**-**	**-**	**-**	**-**	**-**	**1**	**1**	**2**
**rs3802842**	**C11orf53**	**-**	**-**	**-**	**1**	**1**	**-**	**-**	**-**	**2**
**rs4779584**	**GREM1**	**-**	**-**	**-**	**1**	**-**	**-**	**-**	**1**	**2**
**rs7758229**	**SLC22A3**	**1**	**-**	**-**	**-**	**-**	**-**	**1**	**1**	**2**
**rs8180040**	**PTPN23**	**-**	**1**	**-**	**-**	**-**	**-**	**-**	**1**	**2**
**rs8180040**	**PTH1R**	**-**	**-**	**-**	**-**	**-**	**-**	**1**	**1**	**2**
**rs8180040**	**SETD2**	**-**	**-**	**-**	**-**	**-**	**1**	**1**	**-**	**2**
**rs9365723**	**SYNJ2**	**1**	**-**	**1**	**-**	**-**	**-**	**-**	**1**	**2**
**rs9929218**	**CDH3**	**-**	**-**	**-**	**1**	**-**	**-**	**1**	**-**	**2**

To provide empirical evidence of the pipeline, we analyzed the gene scores. Genes with higher biological scores were more likely to be nearest to the risk SNP (62.8% for gene score ⩾ 2, 24% for gene score < 2; p < 0.001). Meanwhile, rectal mucosa cells demonstrated significant overlapping proportions with H3K4me3 peaks compared with other cell types.

Finally, we evaluated the potential role of genetics in relation to drug discovery for the treatment of CRC. Hypergeometric distribution tests were used for overlap statistical analyses. We obtained 11303 genes pairs from curated PPI databases. We obtained 871 drug target genes corresponding to approved, in clinical trials or experimental drugs for human diseases (S14 Table). For the sake of calculation reliability, only CRC drugs in the first line therapy were involved in the study. Eight target genes of approved CRC drugs were included (S15 Table).

**Fig 2:**
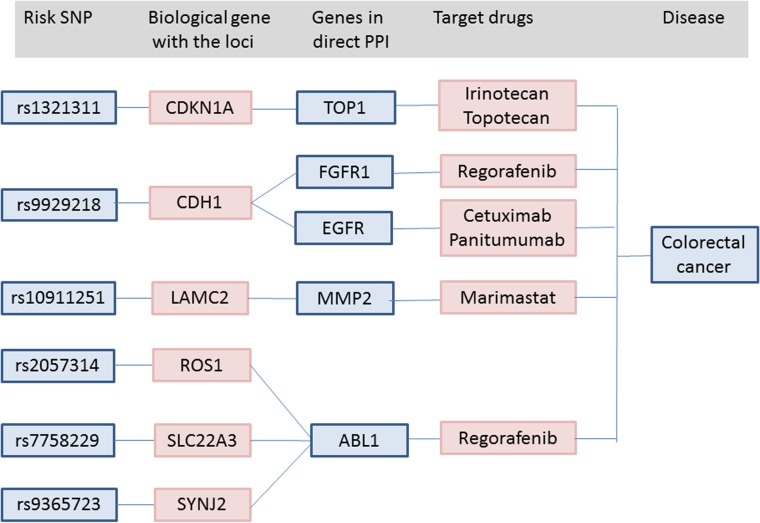
Summary of connections between risk SNPs, biological candidate genes from each risk locus, genes from the PPI network and approved CRC drugs. Black lines indicate connections.

Thirty-one biological CRC risk genes overlapped with 533 genes from the expanded PPI network (S2 Fig. and S3 Fig.). We found an overlap of 5/8 drug target genes of approved CRC drugs (5/8 vs 70/781, 12.09-fold enrichment, P = 0.00013). All 871 drug target genes (regardless of disease indication) overlapped with 70 genes from the PPI network, which suggested that the enrichment was 1.55-fold higher than that expected by chance alone (p = 0.00012), but less by 7.78-fold when compared with currently approved CRC drugs (p = 1.78 × 10^–5^). Examples of approved CRC therapies identified by this analysis included irinotecan, regorafenib, and cetuximab (Fig. 2).

**Fig 3:**
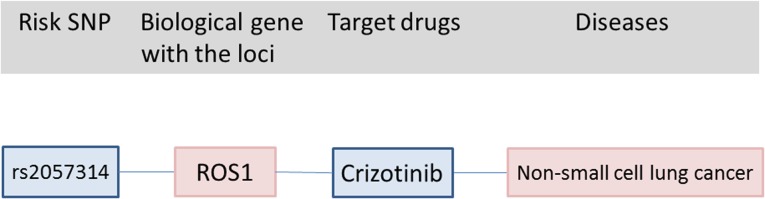
Connections between risk SNP, biological CRC genes and drugs indicated for other diseases.

**Fig 4:**
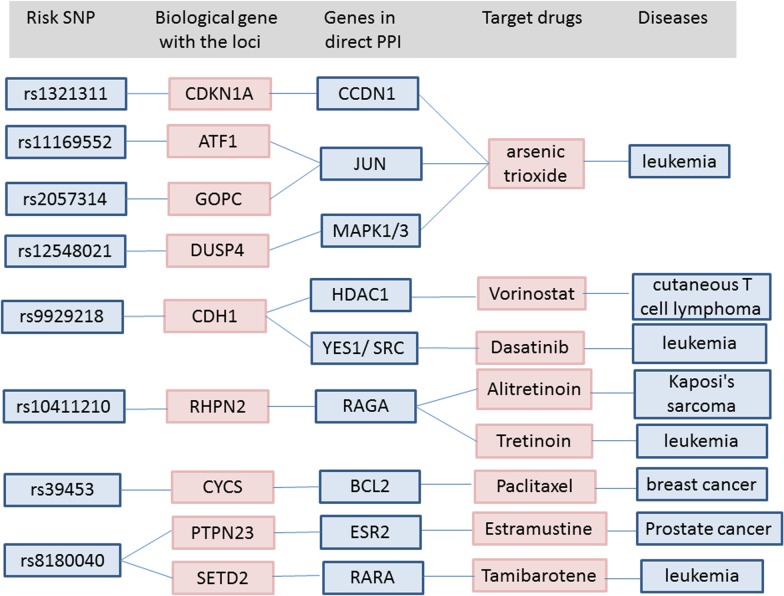
Detailed summary of the connections between risk SNPs, biological candidate genes from each risk locus, genes from the PPI network and drugs indicated for other diseases.

Correlation of approved drugs for other diseases with biological CRC risk gene was also assessed. An example of drug repositioning (Fig. 3) is the use of crizotinib, an approved drug for non-small cell lung cancer for the treatment of CRC [16]. Arsenic trioxide vrinostat, dasatinib, estramustine, and tamibarotene are all promising drugs for the treatment of CRC (Fig. 4).

## Discussion

GWAS have identified innumerable disease-associated genetic variants. However, significant obstacles have hampered our ability to identify genes affected by causal variants and in elucidating the mechanism by which genotype influences phenotype.

Most reports have simply implicated the nearest gene to a GWAS hit without substantial evidence[1]. This study prioritized the most likely target genes. A total of 31 biological CRC risk genes were identified. Although biological CRC risk genes are more likely to be causal genes, this still needs confirmation by basic molecular studies using advanced technologies. Edwards et al provided a pipeline for follow-up studies, which includes fine mapping of risk SNPs, prioritization of putative functional SNPs, and in vitro and in vivo experimental verification of predicted molecular mechanisms for identifying the targeted genes[1].

GWAS is criticized for their lack of clinical translation because of the size of the effect. However, individual small effect sizes do not necessarily preclude clinical utility. Sanseau et al proposed the use of GWAS for drug repositioning, which is regarded as a promising strategy in translational medicine. In a study investigating 3-hydroxy-3-methyl-glutaryl coenzyme A, a well-known cholesterol-lowering medication, SNPs within this gene were unambiguously associated with low-density lipopolysaccharide cholesterol levels in the GWAS data. [6] Their study included all GWAS-associated genes that were selected from the GWAS catalog, without achievement about CRC drugs.

In the present study, we focused on repurposing drugs for CRC based on prioritization of candidate genes in the GWAS-identified loci. For example, crizotinib, arsenic trioxide, vrinostat, dasatinib, estramustine, and tamibarotene are also promising repurposed drugs for CRC. Although further investigations are necessary to confirm the results of this study, we opine that these target drugs selected could be promising drug candidates in the treatment of CRC.

GWAS data is useful in providing insights into the biology of diseases, but may also translate these leads into profitable opportunities in drug development. However, GWAS data does not provide detailed pathophysiological information; hence, the newly identified uses of old drugs may possibly be side effects[23]. Successful repurposing of a drug entails the combination of results from published literature, and clinical research.

Although there were a number of positive aspects from this study, there were some limitations as well. Firstly, data of the PBMC study was used for *cis*-eQTL analysis. Although eQTLs identified from one tissue type may be a useful surrogate to study the genetics of gene expression in another tissue [24], the use of tissue-specific eQTLs is probably more useful in understanding the pathogenesis of CRC [25]. Secondly, of the 34 cell types investigated, we only observed a significant enrichment of risk SNP with H3K4me3 peaks in rectal mucosa cells. Nevertheless, the enrichment was not significant for the colon mucosa cells.

In this study, we integrated genetic data and statistical analysis, computational approaches, and publicly available large data sets to prioritize candidate genes, and propose new targets for CRC drug treatments. We believe that target genes and drugs selected by this approach could be promising leads in the development of candidate drugs for the treatment of CRC, although, further investigations are warranted for confirmation of these results.

## Supporting Information

S1 Fig Histogram distribution of gene scores. Thirty-five genes with a score of >2 were defined as ‘biological risk genes’.
(TIF)

S2 Fig Overlap of 31 biological genes plus 553 genes in direct PPI with them and drug target genes. We found overlap of 5 genes from the 8 drug target genes of approved CRC drugs (12.09-fold enrichment, p = 1.78 × 10^−5^). All 871 drug target genes (regardless of disease indication) overlapped with 70 genes from the PPI network, indicating a 1.55-fold higher enrichment than expected by chance alone (p = 1.20× 10^−4^); but less than 7.78-fold enrichment compared with CRC drugs (p = 1.30 × 10^−4^).
(TIF)

S3 Fig PPI network of biological CRC risk genes and drug target genes. Pink: drug target genes; Orange: CRC risk genes; Cyan: direct PPI genes in PINA2 database.
(TIF)

S1 Table Summary of 140 candidate genes based on proximity and linkage disequilibrium. (XLSX)

S2 Table Risk SNPs annotated by *annovar*. (XLSX)

S3 Table Missense variant in linkage disequilibrium (r^2^ > 0.8) with risk single nucleotide polymorphisms annotated by *ANNOVAR*. (XLSX)

S4 Table Overlap of colorectal cancer risk single nucleotide polymorphisms with H3K4me3 peaks in cells. (DOCX)

S5 Table *cis*-expression quantitative trait loci of colorectal cancer risk single nucleotide polymorphisms. (XLSX)

S6 Table Genes prioritized by PubMed text mining. (XLSX)

S7 Table Genes prioritized by protein-protein interaction network. (XLSX)

S8 Table Overlap of colorectal cancer risk genes with cancer somatic mutation genes. (XLSX)

S9 Table Genes prioritized by knockout mouse phenotype using hypergeometric distribution test. (DOCX)

S10 Table Genes prioritized by go enrichment analysis. (XLSX)

S11 Table Genes prioritized by KEGG enrichment analysis. (XLSX)

S12 Table Genes prioritized by OMIM enrichment analysis. (XLSX)

S13 Table Correlations of biological candidate gene prioritization criteria. (XLSX)

S14 Table A list of drug target genes. (DOCX)

S15 Table Summary of approved drugs for colorectal cancer and target genes. (DOCX)

## References

[1] Edwards SL, Beesley J, French JD, Dunning AM (2013) Beyond GWASs: illuminating the dark road from association to function. Am J Hum Genet 93: 779–797. 10.1016/j.ajhg.2013.10.012 24210251 PMC3824120

[2] Welter D, MacArthur J, Morales J, Burdett T, Hall P, et al (2014) The NHGRI GWAS Catalog, a curated resource of SNP-trait associations. Nucleic Acids Res 42: D1001–1006. 10.1093/nar/gkt1229 24316577 PMC3965119

[3] Cheung VG, Nayak RR, Wang IX, Elwyn S, Cousins SM, et al (2010) Polymorphic cis- and trans-regulation of human gene expression. PLoS Biol 8. 10.1371/journal.pbio.1000480 PMC2939022 20856902

[4] Closa A, Cordero D, Sanz-Pamplona R, Sole X, Crous-Bou M, et al (2014) Identification of candidate susceptibility genes for colorectal cancer through eQTL analysis. Carcinogenesis 35: 2039–2046. 10.1093/carcin/bgu092 24760461 PMC4146415

[5] Csermely P, Korcsmaros T, Kiss HJ, London G, Nussinov R (2013) Structure and dynamics of molecular networks: a novel paradigm of drug discovery: a comprehensive review. Pharmacol Ther 138: 333–408. 10.1016/j.pharmthera.2013.01.016 23384594 PMC3647006

[6] Sanseau P, Agarwal P, Barnes MR, Pastinen T, Richards JB, et al (2012) Use of genome-wide association studies for drug repositioning. Nat Biotechnol 30: 317–320. 10.1038/nbt.2151 22491277

[7] Cao C, Moult J (2014) GWAS and drug targets. BMC Genomics 15 Suppl 4: S5 10.1186/1471-2164-15-S4-S5 25057111 PMC4083410

[8] Okada Y, Wu D, Trynka G, Raj T, Terao C, et al (2014) Genetics of rheumatoid arthritis contributes to biology and drug discovery. Nature 506: 376–381. 10.1038/nature12873 24390342 PMC3944098

[9] Hiersche M, Ruhle F, Stoll M (2013) Postgwas: advanced GWAS interpretation in R. PLoS One 8: e71775 10.1371/journal.pone.0071775 23977141 PMC3747239

[10] Wang K, Li M, Hakonarson H (2010) ANNOVAR: functional annotation of genetic variants from high-throughput sequencing data. Nucleic Acids Res 38: e164 10.1093/nar/gkq603 20601685 PMC2938201

[11] Trynka G, Raychaudhuri S (2013) Using chromatin marks to interpret and localize genetic associations to complex human traits and diseases. Curr Opin Genet Dev 23: 635–641. 10.1016/j.gde.2013.10.009 24287333 PMC4073234

[12] Trynka G, Sandor C, Han B, Xu H, Stranger BE, et al (2013) Chromatin marks identify critical cell types for fine mapping complex trait variants. Nat Genet 45: 124–130. 10.1038/ng.2504 23263488 PMC3826950

[13] Westra HJ, Peters MJ, Esko T, Yaghootkar H, Schurmann C, et al (2013) Systematic identification of trans eQTLs as putative drivers of known disease associations. Nat Genet 45: 1238–1243. 10.1038/ng.2756 24013639 PMC3991562

[14] Raychaudhuri S, Plenge RM, Rossin EJ, Ng AC, International Schizophrenia C, et al (2009) Identifying relationships among genomic disease regions: predicting genes at pathogenic SNP associations and rare deletions. PLoS Genet 5: e1000534 10.1371/journal.pgen.1000534 19557189 PMC2694358

[15] Rossin EJ, Lage K, Raychaudhuri S, Xavier RJ, Tatar D, et al (2011) Proteins encoded in genomic regions associated with immune-mediated disease physically interact and suggest underlying biology. PLoS Genet 7: e1001273 10.1371/journal.pgen.1001273 21249183 PMC3020935

[16] Forbes SA, Bindal N, Bamford S, Cole C, Kok CY, et al (2011) COSMIC: mining complete cancer genomes in the Catalogue of Somatic Mutations in Cancer. Nucleic Acids Res 39: D945–950. 10.1093/nar/gkq929 20952405 PMC3013785

[17] Eppig JT, Blake JA, Bult CJ, Kadin JA, Richardson JE, et al (2012) The Mouse Genome Database (MGD): comprehensive resource for genetics and genomics of the laboratory mouse. Nucleic Acids Res 40: D881–886. 10.1093/nar/gkr974 22075990 PMC3245042

[18] Huang da W, Sherman BT, Lempicki RA (2009) Systematic and integrative analysis of large gene lists using DAVID bioinformatics resources. Nat Protoc 4: 44–57. 10.1038/nprot.2008.211 19131956

[19] Wishart DS, Knox C, Guo AC, Cheng D, Shrivastava S, et al (2008) DrugBank: a knowledgebase for drugs, drug actions and drug targets. Nucleic Acids Res 36: D901–906. 18048412 10.1093/nar/gkm958 PMC2238889

[20] Liu X, Zhu F, Ma X, Tao L, Zhang J, et al (2011) The Therapeutic Target Database: an internet resource for the primary targets of approved, clinical trial and experimental drugs. Expert Opin Ther Targets 15: 903–912. 10.1517/14728222.2011.586635 21619487

[21] Benson AB 3rd, Venook AP, Bekaii-Saab T, Chan E, Chen YJ, et al (2014) Colon cancer, version 3.2014. J Natl Compr Canc Netw 12: 1028–1059. 24994923 10.6004/jnccn.2014.0099

[22] Cowley MJ, Pinese M, Kassahn KS, Waddell N, Pearson JV, et al (2012) PINA v2.0: mining interactome modules. Nucleic Acids Res 40: D862–865. 10.1093/nar/gkr967 22067443 PMC3244997

[23] Wang ZY, Zhang HY (2013) Rational drug repositioning by medical genetics. Nat Biotechnol 31: 1080–1082. 10.1038/nbt.2758 24316641

[24] Cookson W, Liang L, Abecasis G, Moffatt M, Lathrop M (2009) Mapping complex disease traits with global gene expression. Nat Rev Genet 10: 184–194. 10.1038/nrg2537 19223927 PMC4550035

[25] Brown CD, Mangravite LM, Engelhardt BE (2013) Integrative modeling of eQTLs and cis-regulatory elements suggests mechanisms underlying cell type specificity of eQTLs. PLoS Genet 9: e1003649 10.1371/journal.pgen.1003649 23935528 PMC3731231

