# Supplementary figures and images for: Use of Genome-Wide Association Studies for Cancer Research and Drug Repositioning
Source: PLoS One. 2015 Mar 24;10(3):e0116477. doi: 10.1371/journal.pone.0116477 (PMC4372357; doi:10.1371/journal.pone.0116477)

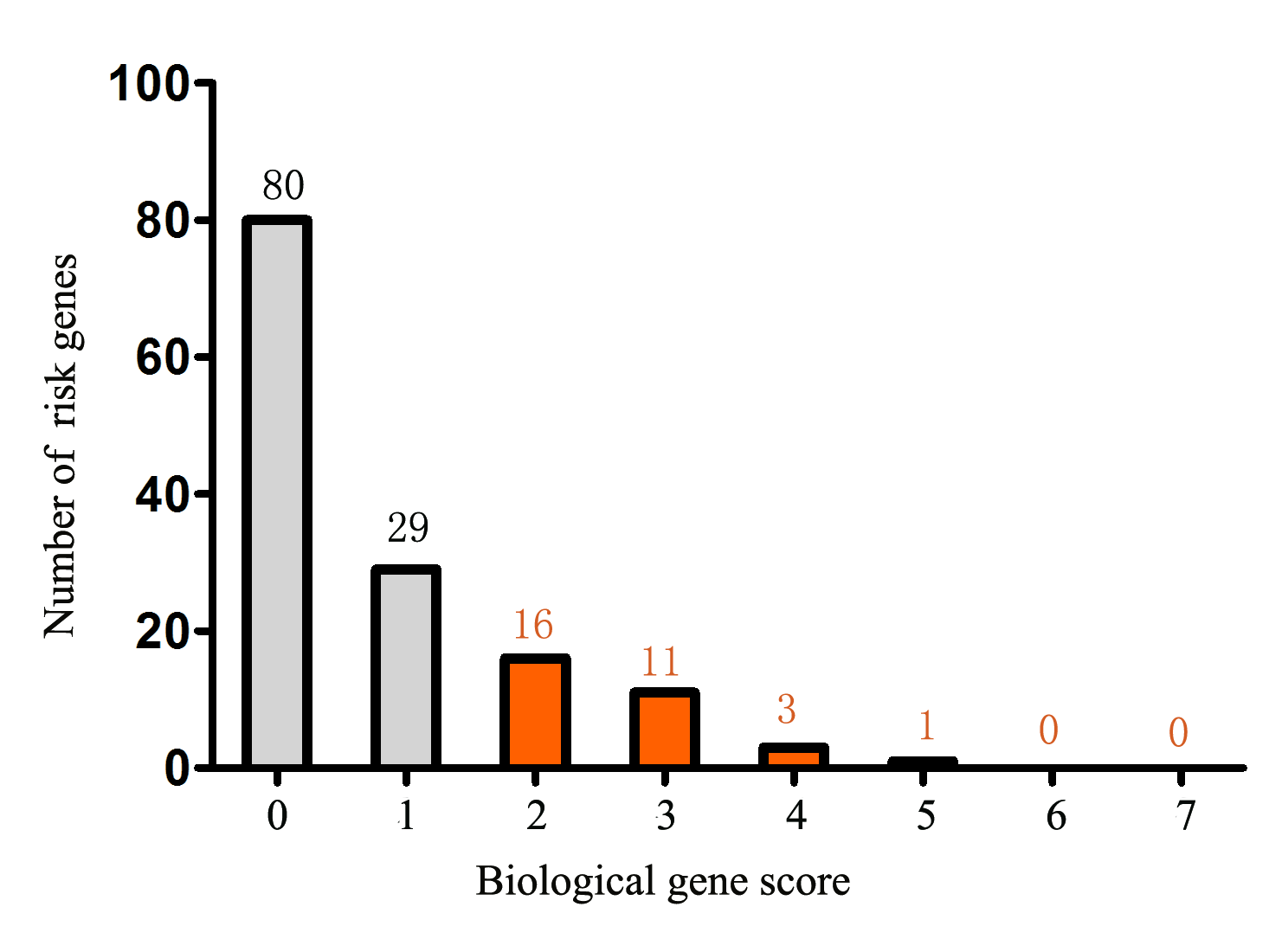

Supplement: S1 Fig — Thirty-five genes with a score of >2 were defined as ‘biological risk genes’. (TIF) [file pone.0116477.s001.tif]

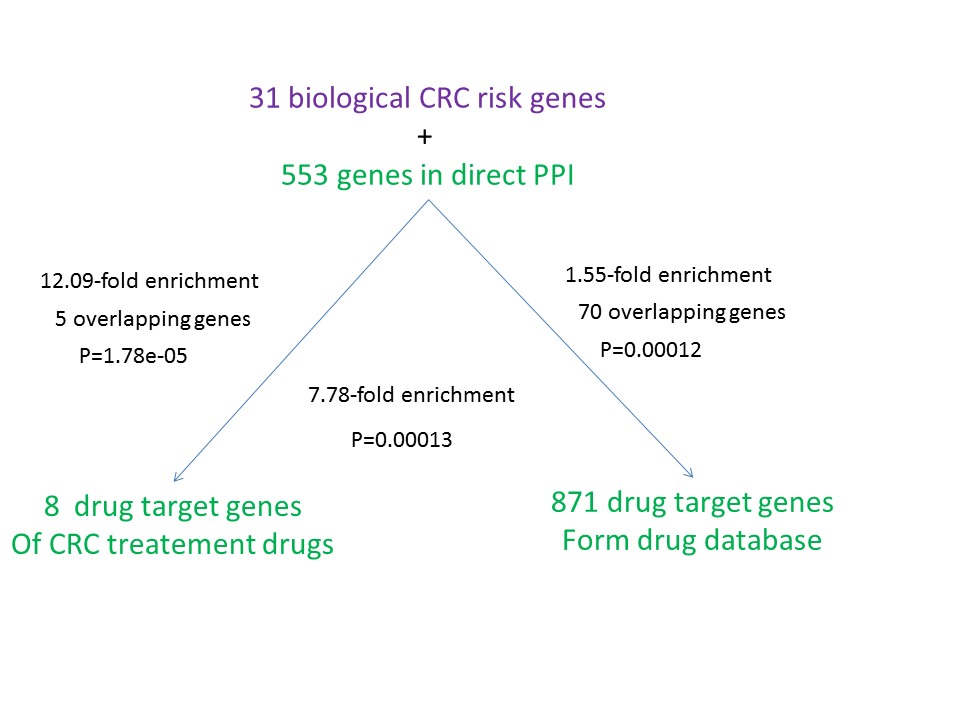

Supplement: S2 Fig — We found overlap of 5 genes from the 8 drug target genes of approved CRC drugs (12.09-fold enrichment, p = 1.78 × 10^−5^). All 871 drug target genes (regardless of disease indication) overlapped with 70 genes from the PPI network, indicating a 1.55-fold higher enrichment than expected by chance alone (p = 1.20× 10^−4^); but less than 7.78-fold enrichment compared with CRC drugs (p = 1.30 × 10^−4^). (TIF) [file pone.0116477.s002.tif]

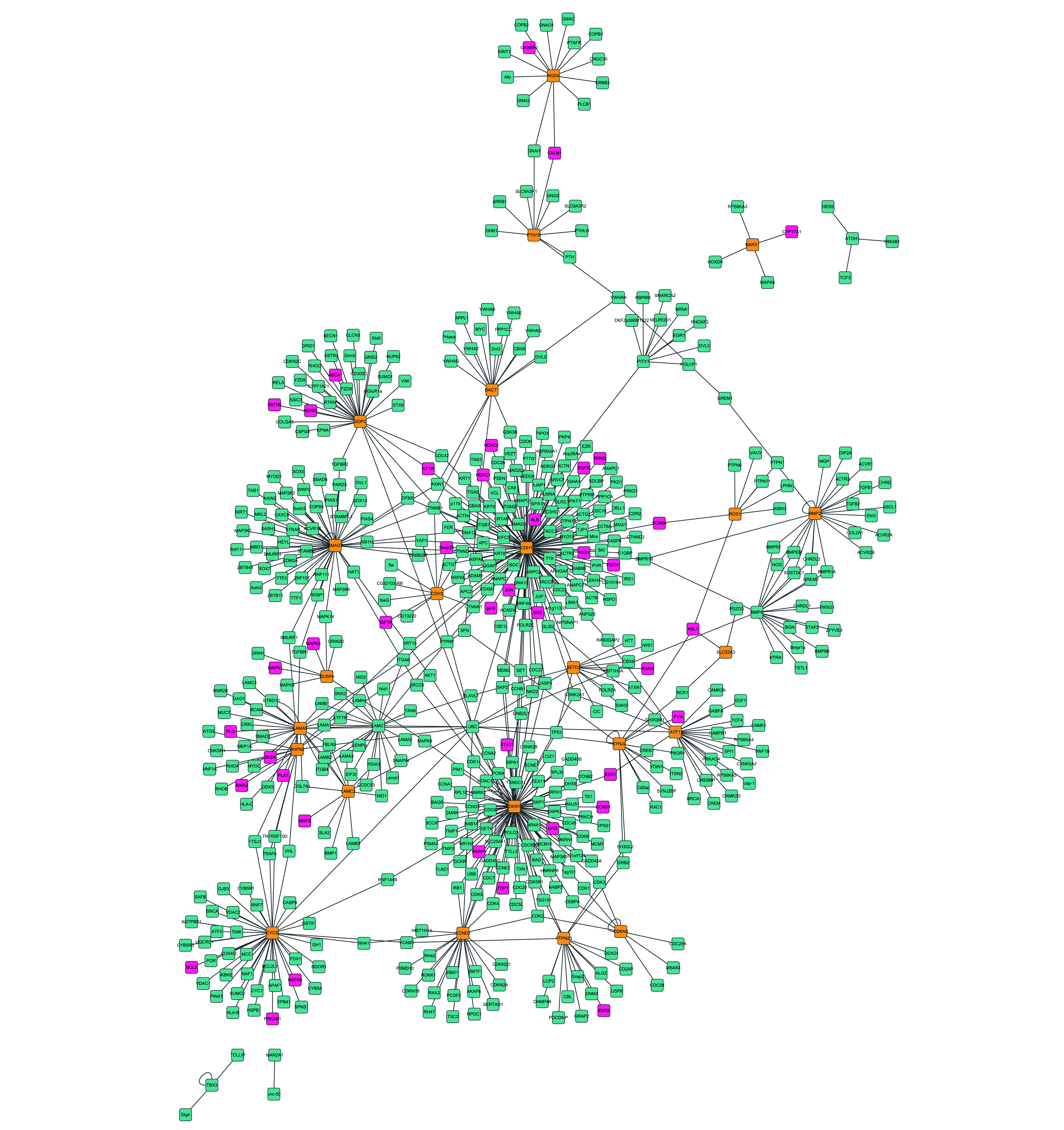

Supplement: S3 Fig — Pink: drug target genes; Orange: CRC risk genes; Cyan: direct PPI genes in PINA2 database. (TIF) [file pone.0116477.s003.tif]
