# Supplementary material for: Use of Genome-Wide Association Studies for Cancer Research and Drug Repositioning
Source: PLoS One. 2015 Mar 24;10(3):e0116477. doi: 10.1371/journal.pone.0116477 (PMC4372357; doi:10.1371/journal.pone.0116477)
Supplement: S4 Table — (DOCX) [file pone.0116477.s007.docx]

**Table S4. Overlap of colorectal cancer risk single nucleotide polymorphisms with H3K4me3 peaks in cells.**

| Tissue EUR | Score | Tissue ASN | Score |
| --- | --- | --- | --- |
| Rectal Mucosa | 0.0001 | Rectal Mucosa | 0.0002 |
| Brain Inferior Temporal Lobe | 0.0687 | Muscle Satellite Cultured Cells | 0.0556 |
| Chondrocytes from Bone Marrow Derived Mesenchymal Stem Cell Cultured Cells | 0.0785 | Chondrocytes from Bone Marrow Derived Mesenchymal Stem Cell Cultured Cells | 0.1333 |
| Duodenum Smooth Muscle | 0.0854 | Brain Anterior Caudate | 0.1407 |
| Stomach Mucosa | 0.1702 | Duodenum Smooth Muscle | 0.1416 |
| Brain Cingulate Gyrus | 0.2354 | Brain Inferior Temporal Lobe | 0.1803 |
| Mesenchymal Stem Cell Derived Adipocyte Cultured Cells | 0.3365 | Brain Cingulate Gyrus | 0.1828 |
| CD8 Naive Primary Cells | 0.3431 | Bone Marrow Derived Mesenchymal Stem Cell Cultured Cells | 0.1947 |
| Adult Liver | 0.3772 | Colon Smooth Muscle | 0.1950 |
| Skeletal Muscle | 0.3807 | Mesenchymal Stem Cell Derived Adipocyte Cultured Cells | 0.2129 |
| Bone Marrow Derived Mesenchymal Stem Cell Cultured Cells | 0.3962 | Adipose Derived Mesenchymal Stem Cell Cultured Cells | 0.2236 |
| Muscle Satellite Cultured Cells | 0.3969 | Treg Primary Cells | 0.3772 |
| Pancreatic Islets | 0.4053 | Stomach Mucosa | 0.4096 |
| Treg Primary Cells | 0.4361 | Stomach Smooth Muscle | 0.4741 |
| Colon Smooth Muscle | 0.4384 | CD34 Primary Cells | 0.5391 |
| CD3 Primary Cells | 0.5134 | Rectal Smooth Muscle | 0.5757 |
| CD4 Memory Primary Cells | 0.6119 | CD3 Primary Cells | 0.6184 |
| Rectal Smooth Muscle | 0.6580 | CD19 Primary Cells | 0.6184 |
| Adipose Nuclei | 0.6589 | Brain Hippocampus Middle | 0.6303 |
| CD34 Cultured Cells | 0.6987 | Skeletal Muscle | 0.6806 |
| CD19 Primary Cells | 0.7024 | Adult Kidney | 0.6993 |
| Adult Kidney | 0.7284 | Colonic Mucosa | 0.7143 |
| CD4 Naive Primary Cells | 0.7875 | Duodenum Mucosa | 0.7280 |
| CD34 Primary Cells | 0.7893 | CD4 Memory Primary Cells | 0.7854 |
| Stomach Smooth Muscle | 0.7900 | CD8 Naive Primary Cells | 0.7888 |
| Brain Anterior Caudate | 0.8006 | Pancreatic Islets | 0.7891 |
| Brain Mid Frontal Lobe | 0.8454 | CD34 Cultured Cells | 0.7932 |
| Adipose Derived Mesenchymal Stem Cell Cultured Cells | 0.8540 | Mobilized CD34 Primary Cells | 0.8167 |
| Duodenum Mucosa | 0.8619 | Adipose Nuclei | 0.8174 |
| Colonic Mucosa | 0.8639 | Adult Liver | 0.8278 |
| Brain Hippocampus Middle | 0.8852 | Brain Mid Frontal Lobe | 0.8413 |
| CD8 Memory Primary Cells | 0.9179 | CD8 Memory Primary Cells | 0.8848 |
| Mobilized CD34 Primary Cells | 0.9239 | Brain Substantia Nigra | 0.9372 |
| Brain Substantia Nigra | 0.9394 | CD4 Naive Primary Cells | 0.9441 |
