# Supplementary material for: Use of Genome-Wide Association Studies for Cancer Research and Drug Repositioning
Source: PLoS One. 2015 Mar 24;10(3):e0116477. doi: 10.1371/journal.pone.0116477 (PMC4372357; doi:10.1371/journal.pone.0116477)
Supplement: S9 Table — (DOCX) [file pone.0116477.s012.docx]

**Table S9. Genes prioritized by knockout mouse phenotype using hypergeometric distribution test.**

| Knockout mouse phenotype | No. knock mouse genes with human orhology | No. overlap with risk genes | p value |
| --- | --- | --- | --- |
| craniofacial phenotype | 809 | 12 | 0.0010 |
| cardiovascular system phenotype | 1693 | 19 | 0.0013 |
| hearing/vestibular/ear phenotype | 420 | 7 | 0.0043 |
| limbs/digits/tail phenotype | 618 | 9 | 0.0043 |
| digestive/alimentary phenotype | 1022 | 12 | 0.0082 |
| skeleton phenotype | 1213 | 13 | 0.0137 |
| embryogenesis phenotype | 1237 | 13 | 0.0162 |
| respiratory system phenotype | 999 | 10 | 0.0431 |
| endocrine/exocrine gland phenotype | 1266 | 12 | 0.0448 |
| adipose tissue phenotype | 543 | 6 | 0.0535 |
| nervous system phenotype | 2311 | 19 | 0.0567 |
| reproductive system phenotype | 1317 | 12 | 0.0593 |
| homeostasis/metabolism phenotype | 2850 | 22 | 0.0779 |
| integument phenotype | 1257 | 11 | 0.0876 |
| renal/urinary system phenotype | 866 | 8 | 0.0913 |
| normal phenotype | 1440 | 12 | 0.1077 |
| muscle phenotype | 1062 | 9 | 0.1270 |
| mortality/aging | 3162 | 23 | 0.1377 |
| other phenotype | 202 | 2 | 0.1381 |
| liver/biliary system phenotype | 874 | 7 | 0.1869 |
| cellular phenotype | 2369 | 17 | 0.2159 |
| growth/size/body phenotype | 2546 | 18 | 0.2316 |
| vision/eye phenotype | 930 | 6 | 0.3864 |
| pigmentation phenotype | 247 | 1 | 0.4745 |
| tumorigenesis | 722 | 4 | 0.5004 |
| taste/olfaction phenotype | 109 | 0 | 0.5073 |
| behavior/neurological phenotype | 1991 | 10 | 0.7834 |
| immune system phenotype | 2279 | 11 | 0.8555 |
| hematopoietic system phenotype | 2095 | 9 | 0.9154 |
