# Supplementary material for: Use of Genome-Wide Association Studies for Cancer Research and Drug Repositioning
Source: PLoS One. 2015 Mar 24;10(3):e0116477. doi: 10.1371/journal.pone.0116477 (PMC4372357; doi:10.1371/journal.pone.0116477)
Supplement: S14 Table — (DOCX) [file pone.0116477.s017.docx]

**Table S14. A list of drug target genes..**

| **A list of drug target genes..** | | | | | | |
| --- | --- | --- | --- | --- | --- | --- |
| TNFRSF4 | ADORA1 | RAF1 | GLRB | PDE7B | TOP1MT | TPH1 |
| SCNN1D | REN | THRB | NPY1R | IFNGR1 | DGAT1 | CAT |
| MMP23A | AVPR1B | RARB | NPY5R | GRM1 | SLC52A2 | CHRM4 |
| GABRD | IKBKE | TOP2B | GLRA3 | ESR1 | JAK2 | F2 |
| MTOR | HSD11B1 | CCR4 | SLC25A4 | OPRM1 | TEK | FOLH1 |
| EPHA2 | KCNK2 | SCN5A | TLR3 | PLG | CNTFR | MS4A2 |
| HTR6 | ESRRG | SCN10A | KLKB1 | PDE10A | SIGMAR1 | MS4A1 |
| PLA2G2A | TGFB2 | SCN11A | F11 | PSMB1 | IL11RA | PTGDR2 |
| ALPL | PARP1 | VIPR1 | MTNR1A | HDAC9 | NPR2 | PGA3 |
| HTR1D | GGPS1 | CCR1 | SLC6A3 | IL6 | ANXA1 | FADS1 |
| CNR2 | MTR | CCR3 | SRD5A1 | CRHR2 | RFK | FADS2 |
| CD52 | RYR2 | CCR2 | PRLR | GHRHR | SYK | FTH1 |
| PTAFR | CHRM3 | CCR5 | IL7R | PDE1C | FBP1 | CHRM1 |
| OPRD1 | TPO | PTH1R | PTGER4 | BLVRA | GABBR2 | SLC22A6 |
| HCRTR1 | ADAM17 | IMPDH2 | PRKAA1 | GCK | GRIN3A | SLC22A8 |
| LCK | RRM2 | CACNA2D2 | GHR | NPC1L1 | PPP3R2 | PRDX5 |
| HDAC1 | ODC1 | GRM2 | IL6ST | PPIA | ABCA1 | SLC22A11 |
| PSMB2 | NTSR2 | TLR9 | PDE4D | RAMP3 | TXN | SLC22A12 |
| CSF3R | APOB | TNNC1 | HTR1A | ADCY1 | UGCG | PYGM |
| KCNQ4 | KCNK3 | PRKCD | CDK7 | DDC | ALAD | CPT1A |
| MPL | XDH | TKT | CARTPT | EGFR | POLE3 | CCND1 |
| SLC6A9 | SRD5A2 | CACNA1D | HMGCR | VKORC1L1 | TLR4 | FOLR3 |
| AKR1A1 | SLC8A1 | UBA3 | F2R | HSPB1 | C5 | FOLR2 |
| FAAH | PRKCE | HTR1F | PDE8B | HGF | PTGS1 | P2RY2 |
| CPT2 | EPAS1 | PROS1 | DHFR | CACNA2D1 | CDK9 | SLCO2B1 |
| DIO1 | CALM2 | GABRR3 | CKMT2 | GRM3 | ABL1 | DGAT2 |
| PCSK9 | LHCGR | ATP6V1A | CHD1 | ABCB4 | DBH | GRM5 |
| JUN | FSHR | DRD3 | SLC12A2 | CYP51A1 | RXRA | TYR |
| PDE4B | POLE4 | CD80 | IL13 | CDK6 | EGFL7 | MTNR1B |
| PTGER3 | TACR1 | NR1I2 | CAMLG | CALCR | GRIN1 | PGR |
| PTGFR | GGCX | GSK3B | IL9 | CYP3A4 | TUBB4B | MMP7 |
| S1PR1 | ADRA2B | CD86 | HDAC3 | EPHB4 | CACNA1B | MMP20 |
| AMY2A | IL1R2 | CASR | FGF1 | ACHE | AKR1C2 | MMP27 |
| CSF1 | IL1R1 | ATP2C1 | NR3C1 | SERPINE1 | IL2RA | MMP8 |
| ADORA3 | IL1B | AGTR1 | HTR4 | PIK3CG | PRKCQ | MMP10 |
| KCND3 | SCTR | P2RY12 | ADRB2 | MET | CACNB2 | MMP1 |
| ATP1A1 | PROC | MME | CSF1R | IMPDH1 | ITGB1 | MMP3 |
| CD2 | SMPD4 | IL12A | PDGFRB | SMO | NRP1 | MMP12 |
| HSD3B2 | CXCR4 | SI | GLRA1 | AKR1B1 | RET | MMP13 |
| HSD3B1 | HNMT | BCHE | GRIA1 | CHRM2 | ALOX5 | GUCY1A2 |
| FCGR1B | CACNB4 | GHSR | ADRA1B | AKR1D1 | PPYR1 | ACAT1 |
| GNRHR2 | DPP4 | PSMD2 | GABRB2 | TBXAS1 | MAPK8 | SDHD |
| FCGR1A | SCN3A | CLCN2 | GABRA6 | BRAF | CDK1 | IL18 |
| SV2A | SCN2A | SST | GABRA1 | MGAM | SIRT1 | DRD2 |
| CA14 | SCN1A | FGFR3 | GABRG2 | TPK1 | TACR2 | HTR3B |
| MCL1 | SCN9A | ADRA2C | GABRP | KCNH2 | EIF4EBP2 | HTR3A |
| CTSS | ABCB11 | DRD5 | DRD1 | NOS3 | DDIT4 | NNMT |
| CTSK | PDK1 | CCKAR | HRH2 | ANGPT2 | PLAU | APOC3 |
| NPR1 | ITGA4 | CHRNA9 | FGFR4 | FDFT1 | ADK | BACE1 |
| IL6R | PDE1A | GABRG1 | FLT4 | LPL | VDAC2 | FXYD2 |
| CHRNB2 | ITGAV | GABRA2 | NQO2 | SLC18A1 | KCNMA1 | SCN4B |
| GBA | CALCRL | GABRA4 | SIRT5 | ATP6V1B2 | LIPF | SCN2B |
| FDPS | TFPI | GABRB1 | TPMT | TNFRSF10B | HTR7 | CD3E |
| BGLAP | STAT1 | PDGFRA | ALDH5A1 | TNFRSF10A | IDE | CD3D |
| NTRK1 | PLCL1 | KIT | GABBR1 | ADRA1A | KIF11 | CD3G |
| CD1A | AOX1 | KDR | TUBB | CHRNA2 | CYP26A1 | HSPA8 |
| FCER1A | CD28 | GNRHR | LTA | CLU | ABCC2 | SCN3B |
| CRP | CTLA4 | DCK | TNF | GSR | CHUK | CHEK1 |
| KCNJ10 | PTH2R | ALB | SLC44A4 | ADRB3 | SCD | DCPS |
| PVRL4 | MAP2 | CXCL2 | C4A | FGFR1 | NFKB2 | KCNJ1 |
| FCER1G | CPS1 | HPSE | C4B | IDO1 | CYP17A1 | KCNJ5 |
| NR1I3 | ERBB4 | EIF4E | RXRB | PLAT | ADRA2A | CACNA1C |
| FCGR2A | ATIC | METAP1 | ITPR3 | IKBKB | ADRB1 | KCNA1 |
| FCGR3A | FN1 | ADH1A | PPARD | POLB | PNLIP | KCNA5 |
| FCGR2C | CXCR2 | ADH1B | MAPK14 | VDAC3 | SLC18A2 | VWF |
| FCGR3B | CXCR1 | ADH1C | GLP1R | FNTA | FGFR2 | SCNN1A |
| FCGR2B | GPBAR1 | MTTP | VEGFA | OPRK1 | MMP21 | VAMP1 |
| RXRG | TUBA4A | NFKB1 | PLA2G7 | PDE7A | CALY | CD4 |
| CD247 | PSMD1 | CENPE | MUT | TRPA1 | DRD4 | PTPN6 |
| F5 | HTR2B | TACR3 | IL17A | CA1 | TH | C1S |
| SELP | ALPPL2 | EGF | HTR1B | CA3 | KCNQ1 | M6PR |
| SELE | TRPM8 | PDE5A | CNR1 | CA2 | CHRNA10 | GRIN2B |
| SERPINC1 | RAMP1 | FGF2 | GABRR1 | MMP16 | RRM1 | GUCY2C |
| ABL2 | HDAC4 | SLC7A11 | GABRR2 | RRM2B | MMP26 | PDE3A |
| SOAT1 | IL5RA | IL15 | FYN | ANGPT1 | HBB | KCNJ8 |
| MR1 | ITPR1 | EDNRA | TUBE1 | SQLE | CCKBR | ITPR2 |
| PTGS2 | OXTR | NR3C2 | HDAC2 | MYC | WEE1 | VDR |
| PLA2G4A | SLC6A1 | TLR2 | CTGF | KCNQ3 | CALCA | CACNB3 |
| CACNA1S | HRH1 | FGA | TAAR1 | KCNK9 | KCNJ11 | TUBA1A |
| SYT2 | PPARG | NPY2R | MYB | CYP11B1 | ABCC8 | ASIC1 |
| ACVRL1 | CYP19A1 | PRKCA | TUBB1 | TXNRD1 | SCNN1G | SERPINB2 |
| ITGB7 | APH1B | CACNG1 | HRH3 | ACACB | SCNN1B | MADCAM1 |
| RARG | PPIB | SSTR2 | NTSR1 | ALDH2 | PLK1 | ELANE |
| ITGA5 | CYP11A1 | GRIN2C | CHRNA4 | NOS1 | PRKCB | CFD |
| PDE1B | MPI | SPHK1 | KCNQ2 | PRKAB1 | IL4R | KISS1R |
| MMP19 | CHRNA3 | BIRC5 | APP | PLA2G1B | CD19 | GRIN3B |
| CDK2 | CHRNB4 | GAA | GRIK1 | P2RX7 | QPRT | GAMT |
| ERBB3 | PDE8A | GCGR | SOD1 | ORAI1 | MAPK3 | TBXA2R |
| IL23A | IGF1R | FASN | IFNAR2 | HPD | ITGAL | PLIN3 |
| CDK4 | HBA2 | TYMS | IFNAR1 | HCAR2 | VKORC1 | PTPRS |
| AVPR1A | HBA1 | YES1 | IFNGR2 | HCAR3 | SLC5A2 | CD70 |
| IFNG | SSTR5 | MC2R | GART | MMP17 | MMP2 | C3 |
| MDM2 | CACNA1H | ROCK1 | KCNE1 | POLE | SLC6A2 | INSR |
| METAP2 | NOXO1 | CDH2 | CBS | FLT3 | CES1 | DNMT1 |
| LTA4H | MMP25 | TTR | PDXK | FLT1 | SLC12A3 | ICAM1 |
| NR1H4 | ABAT | FECH | COMT | ALOX5AP | CETP | PDE4A |
| IGF1 | GRIN2A | MC4R | SERPIND1 | TNFSF11 | MMP15 | EPOR |
| PAH | ABCC1 | BCL2 | MAPK1 | CPB2 | CA7 | CACNA1A |
| AKT1 | ITGA2B | MMP24 | GABRQ | HTR2A | AGRP | PTGER1 |
| GABRB3 | CRHR1 | SRC | SLC6A8 | CYSLTR2 | SLC12A4 | JAK3 |
| GABRA5 | ITGB3 | TGM2 | AVPR2 | MLNR | PDF | PDE4C |
| GABRG3 | NPEPPS | TOP1 | F8 | EDNRB | NQO1 | SCN1B |
| OCA2 | NGFR | ADA | HSP90AA1 | TPP2 | DHODH | HPN |
| CHRFAM7A | CACNA1G | TNNC2 | MARK3 | TNFSF13B | DPEP1 | CD22 |
| CHRNA7 | MPO | MMP9 | CKB | F7 | TUBB3 | FFAR1 |
| RYR3 | TUBD1 | SLC12A5 | TUBG1 | F10 | SERPINF1 | ATP4A |
| CHRM5 | RPS6KB1 | CD40 | RAMP2 | TEP1 | TRPV3 | RYR1 |
| MAP1A | CA4 | PTGIS | AOC3 | APEX1 | TRPV1 | BLVRB |
| CKMT1A | ACE | PTPN1 | SNAP25 | PNP | TP53 | EGLN2 |
| SLC12A1 | SCN4A | MC3R | SSTR4 | MMP14 | VAMP2 | RAB4B-EGL |
| HDC | APOH | AURKA | BCL2L1 | PSMB5 | AURKB | CEACAM3 |
| HIF1A | CACNB1 | KLK2 | CYSLTR1 | BCL2L2 | ADORA2B | PLAUR |
| ESR2 | ERBB2 | CD33 | GLA | LTB4R | SHMT1 | KCNN4 |
| PGF | THRA | PRKCG | HTR2C | CTSG | KCNJ12 | CKM |
| TSHR | RARA | AURKC | AGTR2 | SSTR1 | NOS2 | CALM3 |
| CALM1 | TOP2A | FKBP1A | SLC25A5 | POLE2 | SLC6A4 | PTGIR |
| SERPINA6 | STAT3 | SMOX | XIAP | PTGDR | CCL2 | GRIN2D |
| SERPINA1 | NAGLU | ADRA1D | GPR119 | PTGER2 | MMP28 | FTL |
| BDKRB2 | HSD17B1 | SLC23A2 | HPRT1 | F9 | GABRE | GABRA3 |
| MMP11 | SSTR3 | CPT1B | SMS | CSF2RB | TSPO | TLR7 |
| MIF | PDXP | CHKB-CPT1B | POLA1 | IL2RB | PPARA | TLR8 |
| ADORA2A | PLA2G6 | CSF2RA | MAOA | HMOX1 | MAPK12 | GLRA2 |
| CHEK2 | CACNA1I | IL3RA | MAOB | CYP2D6 | STS | ACE2 |
| SLC5A1 | MCHR1 | SLC25A6 | PORCN | HDAC6 | CACNA1F | GRPR |
| AR | IL2RG | CXCR3 |  |  |  |  |
