# Supplementary material for: Use of Genome-Wide Association Studies for Cancer Research and Drug Repositioning
Source: PLoS One. 2015 Mar 24;10(3):e0116477. doi: 10.1371/journal.pone.0116477 (PMC4372357; doi:10.1371/journal.pone.0116477)
Supplement: S15 Table — (DOCX) [file pone.0116477.s018.docx]

**Table S15. Summary of approved drugs for colorectal cancer and target genes.**

| **Drug category** | **Generic name** | **Target gene** |
| --- | --- | --- |
| fluorouracil | \| 5-Fluorouraci \| \| --- \| \| Capecitabine \| \| S1 \| | \| thymidylate synthase \| \| --- \| \| thymidylate synthase \| \| thymidylate synthase; dihydropyrimidine dehydrogenase \| |
| camptothecin | Irinotecan  Topotecan | topoisomerase 1  topoisomerase 1 |
| platin | cisplatin  carboplatin  oxaliplatin | deoxyribonucleic acid |
| molecular targeted drug | cetuximab  bevacizumab  panitumumab  Nimotuzumab | EGFR |
| others | pemetrexed  raltitrexed  arimastat  Regorafenib | thymidylate synthase;dihydrofolate reductase;glycinamide ribonucleotide formyltransferase  thymidylate synthase.  MMP2  FGFR1；ABL1;RET, VEGFR1, VEGFR2, VEGFR3, KIT, PDGFR-alpha, PDGFR-beta, FGFR1, FGFR2,TIE2, DDR2, TrkA, Eph2A, RAF-1, BRAF, BRAFV600E , SAPK2, PTK5 |
